# Global research activity on mental health literacy

**DOI:** 10.1186/s43045-021-00125-5

**Published:** 2021-09-02

**Authors:** Waleed M. Sweileh

**Affiliations:** Department of Physiology and Pharmacology/Toxicology, College of Medicine and Health Sciences, An-Najah National University, Nablus, Palestine

**Keywords:** Mental health, Literacy, Bibliometric, Research, High-income countries

## Abstract

**Background:**

Evidence showed that mental health literacy reduces stigma and promotes help-seeking intentions. This study provides a bibliometric analysis of global research activity on mental health literacy. A bibliometric method was applied using Scopus. The term “mental health literacy” was searched in the title, abstract, and author keywords for the study period from 1900 to 2019. Conventional bibliometric indicators and mapping were generated.

**Results:**

The search query found 945 documents. The earliest documents were published in 1997. The retrieved documents received an average of 25.3 citations per document and an *h*-index of 67. Authors from 68 different countries participated in publishing the retrieved documents. Australia ranked first (n=354, 37.5%) followed distantly by the USA (n=172, 18.2%). In total, 362 different journals participated in publishing the retrieved documents. The *Australian and New Zealand Journal of Psychiatry* (n=43, 11.8%) ranked first followed by the *BMC Psychiatry* (n=40, 11.0%). Documents published in the *BMC Psychiatry* journal received the highest number (60.4) of citations per document. In total, 3906 authors participated in publishing the retrieved documents. The average number of authors per document was 4.1. Jorm, A.F ranked first (n=96, 10.2%). Data analysis indicated that the *University of Melbourne* (n=136, 14.1%) ranked first in the number of publications.

**Conclusions:**

Literature on “mental health literacy” is growing rapidly mainly in high-income countries. Research collaboration between active countries and low- and middle-income countries is important since many developing countries lack expertise and the infrastructure for mental health literacy research.

## Background

Almost two decades ago, a group of Australian scholars (Jorm et al., 1997) coined the term “mental health literacy” (MHL) to refer to “knowledge and beliefs about mental disorders which aid their recognition, management, or prevention. Mental health literacy includes the ability to recognize specific disorders; knowing how to seek mental health information; knowledge of risk factors and causes, of self-treatments, and of professional help available; and attitudes that promote recognition and appropriate help-seeking” [1]. This definition was an extension of the term “health literacy” (HL) defined as “the ability to gain access to, understand, and use information in ways which promote and maintain good health” [2]. The definition of MHL coined by Jorm et al. included seven components: “(1) the ability to recognize specific disorders or different types of psychological distress, (2) knowledge and beliefs about risk factors and causes, (3) knowledge and beliefs about self-help interventions, (4) knowledge and beliefs about professional help available, (5) attitudes which facilitate recognition and appropriate help-seeking, (6) knowledge of how to seek mental health information, and (7) early recognition, prevention, and mental health first aid skills” [1]. These seven components were revised and the term NHL was redefined to include the following components: “(1) understanding how to obtain and maintain positive mental health, (2) understanding mental disorders and their treatments, (3) decreasing stigma related to mental disorders, and (4) enhancing help-seeking efficacy, knowing when and where to seek help, and developing competencies designed to improve one’s mental health care and self-management capabilities” [3]. The definition of MHL is not limited to mental health professionals or people with mental health illnesses. Rather, Jorm et al. emphasized the importance of societal and community knowledge of MHL as a means of promoting mental health care [4].

Research on health literacy showed that HL is positively and significantly associated with health outcomes [5, 6]. Research on MHL is more recent and is emerging rapidly due to the relatively common mental health disorders [7, 8]. In the USA, surveys of the general population have reported lifetime mental health disorder prevalence rates of almost 50% [9]. Despite the relatively high prevalence of mental health disorders across different cultures, many people with mental health disorders do not receive any sort of therapy [10]. In the WHO world mental health survey, the authors concluded that increasing population mental health literacy is an important endeavor worldwide [11]. Studies suggested that more than half of serious mental illness cases in developed and less developed countries received no treatment in the year before the survey [12]. This is of clinical concern because early treatment has been shown to improve long-term outcomes [13]. The poor health-seeking behaviors among people with mental health problems are related to a wide range of barriers including economic, social, psychological, and physical services [14, 15].

Assessing scientific publications on MHL is important for assessing research trends and research gaps in different countries where mental health care is far from optimum. Bibliometric analysis and data visualization have been widely used tools to measure and evaluate scientific research quantitatively and qualitatively [16–19]. At least five bibliometric studies HL [20–22] have been published, but none was published on MHL.

There are several scientific databases including Web of Science, Scopus, PubMed, and Google Scholar that would bring out the scientific research metrics available in the literature. Scopus database owned by Elsevier is 100% inclusive of PubMed and included twice the number of journals indexed in the Web of Science [23]. The current study aimed to use the Scopus database, which is large and provides metric analytics, to shed light on the scientific publications MHL. The analysis focused on describing the most productive journals, institutions, authors, citations, and countries, as well as the characteristics of the relevant documents.

## Methods

The current study used bibliometric methodology. Gray literature such as government reports and brochures were not included in the analysis. The current study used Scopus to retrieve data. Since no previous bibliometric studies on MHL were previously published, the search query was developed based on the different components listed in the definition of MHL.

The search strategy was designed to retrieve the maximum number of relevant documents. The keyword used in the search query was limited to the term “mental health literacy.” The MHL term was searched in the title/abstract/author keywords. This approach is simple, straightforward, and will retrieve the maximum number of relevant documents. Furthermore, since the term MHL is relatively recent and specific, it is not possible to find false-positive results. The validity of the search query was confirmed by the findings that Jorm, A.F was the top active author with 98 documents.

The search query was limited to documents published in peer-reviewed journals. No language restriction was imposed.

The retrieved documents were exported from the Scopus database to Microsoft Excel. The information exported included document type, number of documents per year, names of journals, countries, authors, citations, institutions, author keywords, titles, and abstracts. Bibliometric indicators were presented as tables of the top ten that are active, for example, the top ten active countries, journals, authors, and institutions. The research growth was presented as a line graph using the Statistical Package for Social Sciences program. The most frequent author keywords and the most frequent terms in titles/abstracts of the retrieved documents were presented as maps using the VOSviewer free online program [24].

In the visualization map, the size of the node is proportional to the frequency of occurrence of the keyword. The VOSviewer maps allow for various types of analysis including visualization of country collaboration, visualization of author collaboration, and citation analysis as well. In bibliometric analysis, the number of citations per document is used for comparative purposes for active journals or authors, or countries. The retrieved documents are also characterized by the Hirsh index (h-index) which is used as a measure of readability and visibility of the retrieved documents with a higher h-index indicating a higher number of interested researchers in the topic.

## Results

The search query found 945 documents. The earliest documents were published in 1997. The authors of the earliest documents were the Australian research group (Jrom et al. 1997) [1, 25]. The retrieved documents were of several types, mainly research articles (n=839, 88.8%) (Table 1). Of the research article, 394 (47.0%) were identified as descriptive while 338 (40.3%) were identified as interventional/experimental ones.

**Table 1:** Types of retrieved documents on mental health literacy

Document type	Frequency	% (N=945)
Article	839	88.8
Review	78	8.3
Note	8	0.8
Letter	7	0.7
Editorial	5	0.5
Conference paper	4	0.4
Short survey	2	0.2
Undefined	2	0.2

**Fig. 1:**
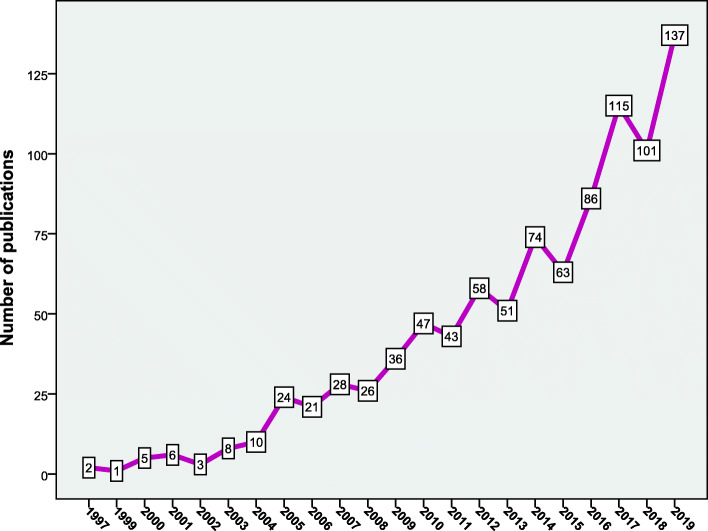
Annual growth of publications on mental health literacy for the study period from 1900 to 2019. A steep increase was observed in the last decade of the study. More than half of the retrieved papers were published from 2015 to 2019

The annual growth of publications remained low from 1997 to 2004 followed by an upward and gradual increase (Fig. 1). The retrieved documents received **23,935** citations, an average of 25.3 citations per document, and an h-index of 67.

Authors from 68 different countries participated in publishing the retrieved documents. Twenty-three (33.8%) countries published 10 or more documents. The total number of documents published by the 23 countries was 888 (94.0%). Australia ranked first (n=354, 37.5%) followed distantly by the USA (n=172, 18.2%) and the UK (n=123, 13.0%). The top ten active countries (Table 2) included countries in Europe, two in North America, two in the Western Pacific region, two in the South-East Asian region, and one in the African region. The list did not include countries from the Eastern Mediterranean region or countries from South America.

**Table 2:** Top ten active countries in publishing research documents on mental health literacy

Rank	Country	Frequency	% (N=945)
**1**	Australia	354	37.5
**2**	USA	172	18.2
**3**	UK	123	13.0
**4**	Canada	93	9.8
**5**	Germany	36	3.8
**6**	Norway	32	3.4
**7**	China	28	3.0
**8**	India	26	2.8
**8**	Japan	26	2.8
**10**	Singapore	23	2.4
**10**	South Africa	23	2.4

In total, 362 different journals participated in publishing the retrieved documents. The *Australian and New Zealand Journal of Psychiatry* (n=43, 11.8%) ranked first followed by the *BMC Psychiatry* (n=40, 11.0%) and *Social Psychiatry and Psychiatric Epidemiology* (n-29, 7.9%). Documents published in the *BMC Psychiatry* journal received the highest number (60.4) of citations per document. The top ten active journals published a total of 267 (28.3%) documents. The top ten active journals (Table 3) were all related to the field of psychiatry except for the *BMC Public Health* journal.

**Table 3:** Top ten active journals in publishing research documents on mental health literacy

Rank	Journal	Frequency	% (N=945)	Number of citations per document
**1**	*Australian And New Zealand Journal Of Psychiatry*	43	11.8	58.2
**2**	*BMC Psychiatry*	40	11.0	60.4
**3**	*Social Psychiatry And Psychiatric Epidemiology*	29	7.9	49.8
**4**	*Psychiatry Research*	27	7.4	13.7
**5**	*Early Intervention In Psychiatry*	23	6.3	25.0
**5**	*International Journal Of Social Psychiatry*	23	6.3	15.9
**7**	*International Journal Of Mental Health Systems*	18	4.9	14.8
**7**	*Journal Of Affective Disorders*	18	4.9	35.5
**7**	*Journal Of Mental Health*	18	4.9	15.4
**10**	*BMC Public Health*	16	4.4	32.2

In total, 3906 authors participated in publishing the retrieved documents. The average number of authors per document was 4.1. There was 83 (8.8%) single-authored documents, 287 (30.4%) two-authored documents, 460 (48.7%) three-authored documents, and 115 (12.2%) multi-authored (≥ 4 authors) documents. Jorm, A.F ranked first (n=96, 10.2%) followed distantly by Reavley, N.J. (n=42, 4.4%). Both have the same institutional affiliation in Australia. The top ten active authors (Table 4) were mainly from Australia.

**Table 4:** Top ten active authors in publishing research documents on mental health literacy

Rank	Author	Frequency	% (N=945)	Affiliation
1	Jorm, A.F.	96	10.2	Australia
2	Reavley, N.J.	42	4.4	Australia
3	Furnham, A.	38	4.0	Norway
4	Christensen, H.	23	2.4	Australia
5	Kutcher, S.	22	2.3	Canada
6	Wei, Y.	20	2.1	Canada
7	Mond, J.M.	19	2.0	Australia
8	Rodgers, B.	16	1.7	Australia
9	Griffiths, K.M.	15	1.6	Australia
10	Hay, P.J.	14	1.5	Australia

Data analysis indicated that the *University of Melbourne* (n=136, 14.1%) ranked first in the number of publications followed by *The Australian National University* (n=55, 5.7%) and the *University College London* (n=41, 4.2%). More than half of the institutions in the top ten active list of institutions were in Australia (Table 5).

**Table 5:** Top ten active authors in publishing research documents on mental health literacy

Rank	Institution/organization	Frequency	% (N=945)	Affiliation
1	*University of Melbourne*	136	14.1	Australia
2	*The Australian National University*	55	5.7	Australia
3	*University College London*	41	4.2	UK
4	*Monash University*	32	3.3	Australia
5	*Western Sydney University*	27	2.8	Australia
6	*La Trobe University*	24	2.5	Australia
6	*Dalhousie University*	24	2.5	Canada
8	*The University of Adelaide*	23	2.4	Australia
9	*The University of Sydney*	22	2.3	Australia
9	*University of New South Wales UNSW Australia*	22	2.3	Australia

**Fig. 2:**
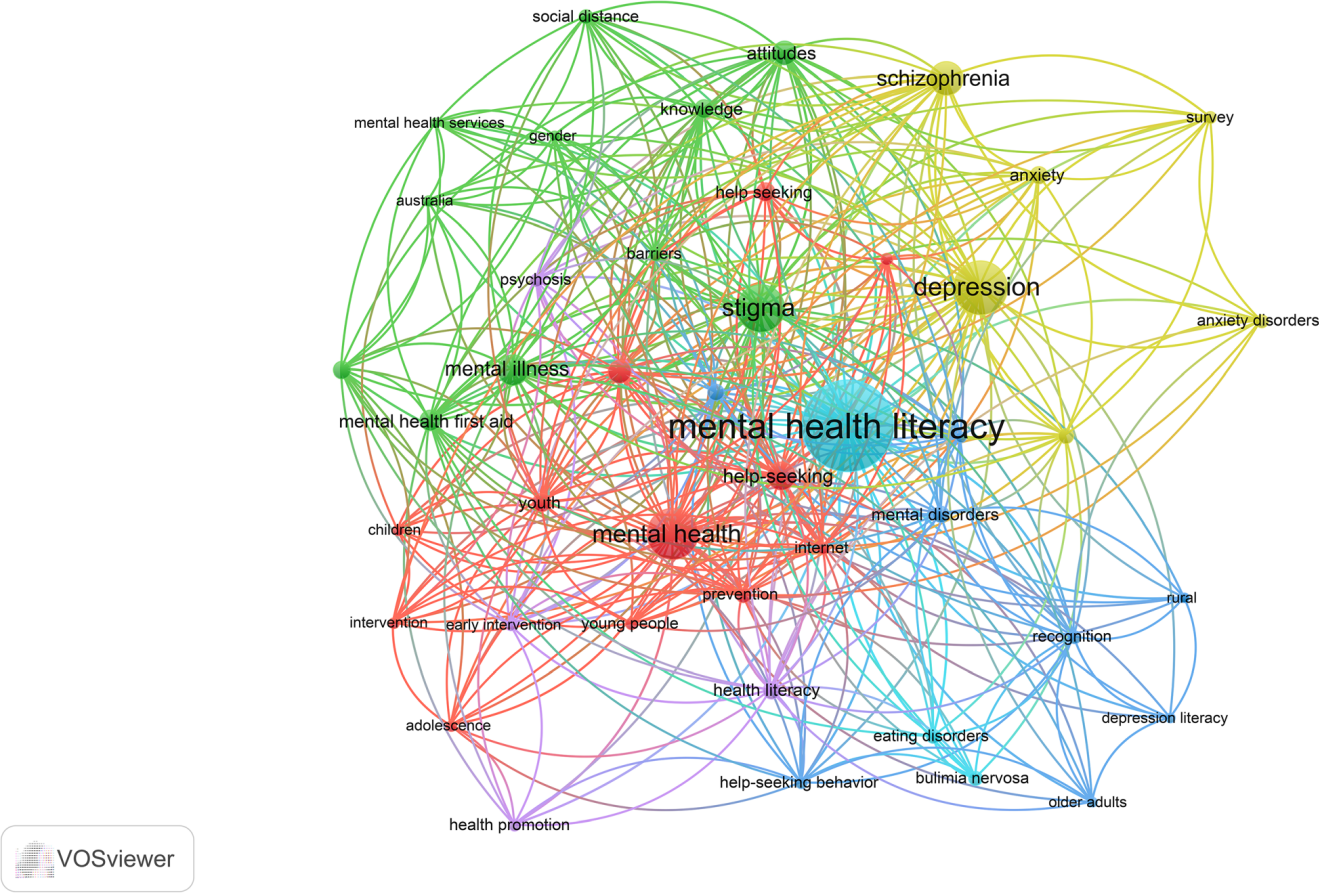
Most frequent author keywords in mental health literacy literature. The map was created using VOSviewer using a minimum frequency of 10 occurrences

Mapping author keywords with minimum occurrences of 30 times (Fig. 2) showed that MHL was the most frequent author keyword followed by “depression,” “stigma,” “schizophrenia,” “help-seeking,” and keywords related to youth/adolescents. Both stigma and help-seeking behaviors were also among the top-cited topics in the retrieved documents. The top ten cited documents (Table 6) included eight research articles and two review articles [1, 4, 11, 26–32]. The highest number of citations achieved was 1019. Two articles achieved this number. When the number of citations was standardized by the year of publication of the article, the highest number of citations per year (n=102) was achieved by a systematic review article about barriers and facilitators to mental health help-seeking in young people.

**Table 6:** Top ten cited documents on mental health literacy

Rank	Authors	Title	Year	Source title	Cited by
1	Gulliver, A., Griffiths, K.M., Christensen, H.	Perceived barriers and facilitators to mental health help-seeking in young people: A systematic review	2010	*BMC Psychiatry*	1019
2	Jorm, A.F., Korten, A.E., Jacomb, P.A., Christensen, H., Rodgers, B., Pollitt, P.	‘Mental health literacy’: A survey of the public's ability to recognise mental disorders and their beliefs about the effectiveness of treatment	1997	*Medical Journal of Australia*	1019
3	Jorm, A.F.	Mental health literacy: Public knowledge and beliefs about mental disorders	2000	*British Journal of Psychiatry*	921
4	Jorm, A.F.	Mental health literacy; empowering the community to take action for better mental health	2012	*American Psychologist*	498
5	Schomerus, G., Schwahn, C., Holzinger, A., Corrigan, P.W., Grabe, H.J., Carta, M.G., Angermeyer, M.C.	Evolution of public attitudes about mental illness: A systematic review and meta-analysis	2012	*Acta Psychiatrica Scandinavica*	448
6	Corrigan, P.W., Druss, B.G., Perlick, D.A.	The impact of mental illness stigma on seeking and participating in mental health care	2014	*Psychological Science in the Public Interest, Supplement*	381
7	Andrade, L.H., Alonso, J., Mneimneh, Z., Wells, J.E., Al-Hamzawi, A., Borges, G., Bromet, E., Bruffaerts, R., De Girolamo, G., De Graaf, R., Florescu, S., Gureje, O., Hinkov, H.R., Hu, C., Huang, Y., Hwang, I., Jin, R., Karam, E.G., Kovess-Masfety, V., Levinson, D., Matschinger, H., O'Neill, S., Posada-Villa, J., Sagar, R., Sampson, N.A., Sasu, C., Stein, D.J., Takeshima, T., Viana, M.C., Xavier, M., Kessler, R.C.	Barriers to mental health treatment: Results from the WHO World Mental Health surveys	2014	*Psychological Medicine*	319
8	Pinfold, V., Toulmin, H., Thornicroft, G., Huxley, P., Farmer, P., Graham, T.	Reducing psychiatric stigma and discrimination: Evaluation of educational interventions in UK secondary schools	2003	*British Journal of Psychiatry*	302
9	Kelly, C.M., Jorm, A.F., Wright, A.	Improving mental health literacy as a strategy to facilitate early intervention for mental disorders.	2007	*The Medical Journal of Australia*	241
10	Kitchener, B.A., Jorm, A.F.	Mental health first aid training for the public: Evaluation of effects on knowledge, attitudes and helping behavior	2002	*BMC Psychiatry*	224

## Discussion

The current study investigated the evolution and research pattern of MHL using the Scopus database to come out with bibliometric indicators and maps. The number of retrieved documents is relatively small, but the number of publications in this field is growing steadily with a high number of citations suggesting a high degree of visibility.

The current study showed that the term MHL has emerged in the late 1990s by Australian researchers. Research on MHL remained low until 2004 followed by a gradual increase. The growth in the number of publications, an approximately 6-fold increase from 2005 to 2019, indicates the importance of the subject to health policymakers and the general public. The noticeable growth of publications on MHL also indicates the recognition by investigators and experts that there is an underestimation of the global burden of mental illnesses. A study showed that the global burden of mental illness accounts for 32.4% of years lived with disability (YLDs) and 13.0% of disability-adjusted life-years (DALYs) and that the currently used approaches underestimate the burden of mental illness by more than a third [33]. The growth of publications on MHL was also driven by attempts of researchers to carry out cross-cultural differences and comparisons regarding levels of MHL which reflects positively on international strategies and help-seeking behaviors in different cultures [34–36]. The introduction of the term MHL led to several reviews on the definition and public health importance of the newly introduced concept [3, 37, 38]. The growth of research on MHL was also driven to assess the public ability to recognize symptoms of mental illness and to minimize the stigma associated with low MHL [39]. A study has shown that education might decrease depression stigma in specific population groups [14, 40]. Research on MHL as a concept is relatively recent and that is why the vast majority of the recruited research articles were descriptive ones and based on observational design. It is the experimental/interventional research methodology that is used in building strong scientific evidence. Therefore, it is expected that research in the field of MHL will witness the emergence of many interventional conclusive studies to be used in scientific evidence.

The current study indicated that authors from Australia, the USA, and the UK contributed to 616 (64.8%) documents on MHL. The leading role of these three countries in MHL research is justifiable. The modern concept of MHL was coined by Australian researchers [1]. These three countries ranked among the top ten active countries in most health topics [41–44]. Mental health research does not seem to be a top priority in low- and middle-income countries. A 10-year survey study on mental health research found that over 90% of mental health research originated from high-income countries [45]. The authors recommended improving research infrastructure and capacity to conduct and disseminate mental health research in low- and middle-income countries. A recent study indicated that the Arab region which included 5.5 of the global population produced only 1.0% of the global research output of mental health research publications [46]. The authors concluded that Arab mental health researchers face challenges such as “prevalent stigma and low awareness, conflict and war, scarce institutional and funding resources, inadequate publishing opportunities, insufficient training in mental health research, and shortage of reliable and valid assessment tools” [46]. Similar findings were reported from other world regions such as Africa [47].

The current study indicated that the list of top ten active journals included one journal in the field of public health, the *BMC Public Health*. Public health and mental health have evolved at the same time, and the mental health of the population has the same importance as biological health [48]. Integration of mental health into public health through screening of mental illness in primary healthcare and increasing awareness of the public to mental illnesses is important to achieve a healthy population [48]. Increasing public awareness and education about mental illness play an important role in minimizing stigma against people with mental disorders [49, 50]. The integration of mental health issues into public health emerged strongly after the COVID-19 crisis [51, 52]. The current study showed that active authors and institutions were mainly based in Australia. This was not surprising given that the early research on the MHL term was initiated by Australian researchers. The extensive experience of researchers in Australia, the USA, and the UK on MHL need to be transferred to researchers in low- and middle-income countries.

The current study showed that publications on MHL were strongly linked to the keyword “stigma.” There are two dimensions of stigma: public stigma and self-stigma. Public stigma involves negative attitudes of the public toward people with mental illness resulting in social distancing and loss of opportunity [53]. Self-stigma occurs when people with mental disorders internalize the public’s negative attitudes and begin to show behaviors that support the negative perceptions [54]. An important and effective approach regarding public stigma toward mental health illnesses is the educational strategies that aim to generate awareness of the mental illness and boost mental health literacy [55]. Studies have demonstrated that an increase in mental health literacy is correlated with a reduction of stigma. It also reduces the social exclusion of people living with mental illness and can encourage help-seeking behavior [56–58].

The current study showed that the author keyword “depression” was frequently encountered in the retrieved literature. Depression is a global mental health problem with approximately 350 million affected individuals [59]. Major barriers in delivering mental health services for people with depression are social stigma and poor MHL [60]. Several studies in different cultures indicated a certain degree of deficits in MHL among adolescents about depression [60–62]. The poor MHL about depression among young and adolescents should be taken into consideration in the school curriculum and mental health campaigns [62].

The current study has a few limitations. Scopus is not inclusive of all journals. Therefore, documents published in journals unindexed in Scopus were missed. The search query implemented was broad and might have retrieved certain false-positive results.

## Conclusions

Literature on MHL is growing rapidly mainly in high-income countries. Scholars and institutions in Australia have a prominent role in this field. The field of MHL is characterized by a relatively high number of citations indicating a large number of interested readers and scholars. Research on MHL is needed everywhere to improve practice and to design interventional studies at all levels to improve public MHL. Research collaboration between active countries and low- and middle-income countries is important since many developing countries lack expertise and the infrastructure for mental health research.

## Data Availability

All data presented in this manuscript are available on the Scopus database using the search query listed in the methodology section.

## Abbreviation

MHL: Mental health literacy

## References

[1] Jorm AF, Korten AE, Jacomb PA, Christensen H, Rodgers B, Pollitt P (1997). “Mental health literacy”: a survey of the public’s ability to recognise mental disorders and their beliefs about the effectiveness of treatment. Med J Aust.

[2] Commonwealth Department of Health H, Services C (1993). Goals and Targets for Australia’s Health in the Year 2000 and Beyond.

[3] Kutcher S, Wei Y, Coniglio C (2016). Mental health literacy: past, present, and future. Can J Psychiatry.

[4] Jorm AF (2000). Mental health literacy: public knowledge and beliefs about mental disorders. Br J Psychiatry.

[5] Berkman ND, Sheridan SL, Donahue KE, Halpern DJ, Crotty K (2011). Low health literacy and health outcomes: an updated systematic review. Ann Intern Med.

[6] Reisi M, Mostafavi F, Javadzade H, Mahaki B, Tavassoli E, Sharifirad G (2016). Impact of health literacy, self-efficacy, and outcome expectations on adherence to self-care behaviors in iranians with type 2 diabetes. Oman Med J.

[7] Williamson V, Stevelink SA, Greenberg K, Greenberg N (2018). Prevalence of mental health disorders in elderly US military veterans: a meta-analysis and systematic review. Am J Geriatr Psychiatry.

[8] Lee YC, Chatterton ML, Magnus A, Mohebbi M, Le LK, Mihalopoulos C (2017). Cost of high prevalence mental disorders: findings from the 2007 Australian National Survey of Mental Health and Wellbeing. Aust N Z J Psychiatry.

[9] Kessler RC, Chiu WT, Demler O, Merikangas KR, Walters EE (2005). Prevalence, severity, and comorbidity of 12-month DSM-IV disorders in the National Comorbidity Survey Replication. Arch Gen Psychiatry.

[10] Alonso J, Codony M, Kovess V, Angermeyer MC, Katz SJ, Haro JM, De Girolamo G, De Graaf R, Demyttenaere K, Vilagut G (2007). Population level of unmet need for mental healthcare in Europe. Br J Psychiatry.

[11] Andrade LH, Alonso J, Mneimneh Z, Wells JE, Al-Hamzawi A, Borges G, Bromet E, Bruffaerts R, de Girolamo G, de Graaf R (2014). Barriers to mental health treatment: results from the WHO World Mental Health surveys. Psychol Med.

[12] Demyttenaere K, Bruffaerts R, Posada-Villa J, Gasquet I, Kovess V, Lepine JP, Angermeyer MC, Bernert S, de Girolamo G, Morosini P (2004). Prevalence, severity, and unmet need for treatment of mental disorders in the World Health Organization World Mental Health Surveys. JAMA.

[13] Clarke M, Whitty P, Browne S, McTigue O, Kamali M, Gervin M, Kinsella A, Waddington JL, Larkin C, O'Callaghan E (2006). Untreated illness and outcome of psychosis. Br J Psychiatry.

[14] Haugen PT, McCrillis AM, Smid GE, Nijdam MJ (2017). Mental health stigma and barriers to mental health care for first responders: a systematic review and meta-analysis. J Psychiatr Res.

[15] Byrow Y, Pajak R, Specker P, Nickerson A (2020). Perceptions of mental health and perceived barriers to mental health help-seeking amongst refugees: a systematic review. Clin Psychol Rev.

[16] Sweileh WM (2017). Global research trends of World Health Organization’s top eight emerging pathogens. Glob Health.

[17] Sweileh WM (2018). Global research output on HIV/AIDS-related medication adherence from 1980 to 2017. BMC Health Serv Res.

[18] Sweileh WM (2018). Bibliometric analysis of peer-reviewed literature in transgender health (1900 - 2017). BMC Int Health Hum Rights.

[19] Sweileh WM, Al-Jabi SW, Zyoud SH, Sawalha AF (2018). Bibliometric analysis of literature in pharmacy education: 2000-2016. Int J Pharm Pract.

[20] Bankson HL (2009). Health literacy: an exploratory bibliometric analysis, 1997–2007. J Med Lib Assoc.

[21] Shapiro RM (2010). Health literacy: a bibliometric and citation analysis.

[22] Wu Q, Liu Z-J, Cheng L, Sun H-L, Han G-H, Pang X-L (2020). The development of Electronic Health Literacy worldwide: a bibliometric analysis. Med Data Mining.

[23] Falagas ME, Pitsouni EI, Malietzis GA, Pappas G (2008). Comparison of PubMed, Scopus, Web of Science, and Google Scholar: strengths and weaknesses. FASEB J.

[24] van Eck NJ, Waltman L (2010). Software survey: VOSviewer, a computer program for bibliometric mapping. Scientometrics.

[25] Jorm AF, Korten AE, Jacomb PA, Christensen H, Rodgers B, Pollitt P (1997). Public beliefs about causes and risk factors for depression and schizophrenia. Soc Psychiatry Psychiatric Epidemiol.

[26] Schomerus G, Schwahn C, Holzinger A, Corrigan PW, Grabe HJ, Carta MG, Angermeyer MC (2012). Evolution of public attitudes about mental illness: a systematic review and meta-analysis. Acta Psychiatrica Scand.

[27] Corrigan PW, Druss BG, Perlick DA (2014). The impact of mental illness stigma on seeking and participating in mental health care. Psychol Sci Public Interest, Suppl.

[28] Kelly CM, Jorm AF, Wright A (2007). Improving mental health literacy as a strategy to facilitate early intervention for mental disorders. Med J Aust.

[29] Kitchener BA, Jorm AF (2002) Mental health first aid training for the public: Evaluation of effects on knowledge, attitudes and helping behavior. BMC Psychiatry 2(1), 1–6. 10.1186/1471-244X-2-10 10.1186/1471-244X-2-10 PMC130043 12359045

[30] Jorm AF (2012). Mental health literacy; empowering the community to take action for better mental health. Am Psychol.

[31] Gulliver A, Griffiths KM, Christensen H (2010) Perceived barriers and facilitators to mental health help-seeking in young people: a systematic review. BMC Psychiatry 10(1), 1–9. 10.1186/1471-244X-10-113 10.1186/1471-244X-10-113 PMC3022639 21192795

[32] Pinfold V, Toulmin H, Thornicroft G, Huxley P, Farmer P, Graham T (2003). Reducing psychiatric stigma and discrimination: evaluation of educational interventions in UK secondary schools. Br J Psychiatry.

[33] Vigo D, Thornicroft G, Atun R (2016). Estimating the true global burden of mental illness. Lancet Psychiatry.

[34] Altweck L, Marshall TC, Ferenczi N, Lefringhausen K (2015). Mental health literacy: a cross-cultural approach to knowledge and beliefs about depression, schizophrenia and generalized anxiety disorder. Front Psychol.

[35] Liu W, Li YM, Peng Y (2018). Mental health literacy: a cross-cultural study of American and Chinese bachelor of nursing students. J Psychiatr Ment Health Nurs.

[36] Vovou F, Hull L, Petrides KV (2020) Mental health literacy of ADHD, autism, schizophrenia, and bipolar disorder: a cross-cultural investigation. J Ment Health 1–11. 10.1080/09638237.2020.1713999 10.1080/09638237.2020.1713999 31994950

[37] Furnham A, Swami V (2018). Mental health literacy: a review of what it is and why it matters. Int Perspect Psychol.

[38] Spiker DA, Hammer JH (2019). Mental health literacy as theory: current challenges and future directions. J Ment Health.

[39] Jorm AF, Nakane Y, Christensen H, Yoshioka K, Griffiths KM, Wata Y (2005). Public beliefs about treatment and outcome of mental disorders: a comparison of Australia and Japan. BMC Med.

[40] Wang J, Lai D (2008). The relationship between mental health literacy, personal contacts and personal stigma against depression. J Affect Disord.

[41] Sweileh WM (2018). Research trends on human trafficking: a bibliometric analysis using Scopus database. Glob Health.

[42] Sweileh WM (2019). Bibliometric analysis of literature in AIDS-related stigma and discrimination. Transl Behav Med.

[43] Sweileh WM, Al-Jabi SW, Sawalha AF, AbuTaha AS, Zyoud SH (2017). Bibliometric analysis of worldwide publications on antimalarial drug resistance (2006-2015). Malar Res Treat.

[44] Sweileh WM, Al-Jabi SW, Zyoud SH, Shraim NY, Anayah FMA, Sawalha AF, AbuTaha AS (2019). Bibliometric analysis of global publications in medication adherence (1900-2017). Int J Pharm Pract.

[45] Saxena S, Paraje G, Sharan P, Karam G, Sadana R (2006). The 10/90 divide in mental health research: trends over a 10-year period. Br J Psychiatry.

[46] Maalouf FT, Alamiri B, Atweh S, Becker AE, Cheour M, Darwish H, Ghandour LA, Ghuloum S, Hamze M, Karam E, Khoury B, Khoury SJ, Mokdad A, Meho LI, Okasha T, Reed GM, Sbaity E, Zeinoun P, Akl EA (2019). Mental health research in the Arab region: challenges and call for action. Lancet Psychiatry.

[47] Chibanda D, Abas M, Musesengwa R, Merritt C, Sorsdahl K, Mangezi W, Bandawe C, Cowan F, Araya R, Gomo E, Gibson L, Weiss H, Hanlon C, Lund C (2020). Mental health research capacity building in sub-Saharan Africa: the African Mental Health Research Initiative. Glob Ment Health (Camb).

[48] Ramírez KVS, Ortiz AIV, Andrade NAO, Bautista JCP: Mental health as an action field for public health. Mexican J Med Res ICSA 2017, 5(10).

[49] Hampson ME, Watt BD, Hicks RE, Bode A, Hampson EJ (2018). Changing hearts and minds: the importance of formal education in reducing stigma associated with mental health conditions. Health Educ J.

[50] Sukhera J, Chahine S (2016) Reducing mental illness stigma through unconscious bias-informed education. MedEdPublish 5(2). 10.15694/mep.2016.000044

[51] Gyasi RM (2020). COVID-19 and mental health of older Africans: an urgency for public health policy and response strategy. Int Psychogeriatr.

[52] Greenberg N (2020). Mental health of health-care workers in the COVID-19 era. Nat Rev Nephrol.

[53] Corrigan PW, Morris SB, Michaels PJ, Rafacz JD, Rüsch N (2012). Challenging the public stigma of mental illness: a meta-analysis of outcome studies. Psychiatric Serv.

[54] Corrigan PW, Watson AC, Barr L (2006). The self–stigma of mental illness: implications for self–esteem and self–efficacy. J Soc Clin Psychol.

[55] Mendenhall AN, Frauenholtz S (2015). Predictors of mental health literacy among parents of youth diagnosed with mood disorders. Child Fam Soc Work.

[56] Gaiha SM, Sunil GA, Kumar R, Menon S (2014) Enhancing mental health literacy in India to reduce stigma: the fountainhead to improve help-seeking behaviour. J Public Ment Health 146–158

[57] Jung H, von Sternberg K, Davis K (2017). The impact of mental health literacy, stigma, and social support on attitudes toward mental health help-seeking. Int J Ment Health Promot.

[58] Cheng HL, Wang C, McDermott RC, Kridel M, Rislin JL (2018). Self-stigma, mental health literacy, and attitudes toward seeking psychological help. J Couns Dev.

[59] World Health Organization (WHO) (2012). Depression, a hidden burden.

[60] Coles ME, Ravid A, Gibb B, George-Denn D, Bronstein LR, McLeod S (2016). Adolescent mental health literacy: young people’s knowledge of depression and social anxiety disorder. J Adolesc Health.

[61] Loureiro LM, Jorm AF, Mendes AC, Santos JC, Ferreira RO, Pedreiro AT (2013). Mental health literacy about depression: a survey of portuguese youth. BMC Psychiatry.

[62] Melas P, Tartani E, Forsner T, Edhborg M, Forsell Y (2013). Mental health literacy about depression and schizophrenia among adolescents in Sweden. Eur Psychiatry.

